# Untargeted metabolomics of saliva in pregnant women with and without gestational diabetes mellitus and healthy non-pregnant women

**DOI:** 10.3389/fcimb.2023.1206462

**Published:** 2023-07-19

**Authors:** Yueheng Li, Yang Feng, Zhengyan Yang, Zhi Zhou, Dan Jiang, Jun Luo

**Affiliations:** ^1^ Department of Preventive Dentistry, Stomatological Hospital of Chongqing Medical University, Chongqing, China; ^2^ College of Stomatology, Chongqing Medical University, Chongqing, China; ^3^ Chongqing Key Laboratory of Oral Diseases and Biomedical Sciences, Chongqing, China; ^4^ Chongqing Municipal Key Laboratory of Oral Biomedical Engineering of Higher Education, Chongqing, China; ^5^ Chongqing Changshou Health Center for Women and Children, Chongqing, China

**Keywords:** gestational diabetes mellitus, pregnant women, saliva, gingivitis, untargeted metabolomics

## Abstract

**Objective:**

The aim of this study was to compare the differences in salivary metabolites between pregnant women with gestational diabetes mellitus (GDM), healthy pregnant women (HPW), and healthy non-pregnant women (HNPW), and analyze the possible associations between the identified metabolites and gingivitis.

**Method:**

The study included women with GDM (*n* = 9, mean age 28.9 ± 3.6 years, mean gestational age 30.1 ± 3.2 weeks), HPW (*n* = 9, mean age 27.9 ± 3.0 years, mean gestational age 28.6 ± 4.7 weeks), and HNPW (*n* = 9, mean age 27.7 ± 2.1 years). Saliva samples were collected from all participants and were analyzed with LC-MS/MS-based untargeted metabolomic analysis. Metabolite extraction, qualitative and semi-quantitative analysis, and bioinformatics analysis were performed to identify the differential metabolites and metabolic pathways between groups. The identified differential metabolites were further analyzed in an attempt to explore their possible associations with periodontal health and provide evidence for the prevention and treatment of periodontal inflammation during pregnancy.

**Results:**

In positive ion mode, a total of 2,529 molecular features were detected in all samples, 166 differential metabolites were identified between the GDM and HPW groups (89 upregulated and 77 downregulated), 823 differential metabolites were identified between the GDM and HNPW groups (402 upregulated and 421 downregulated), and 647 differential metabolites were identified between the HPW and HNPW groups (351 upregulated and 296 downregulated). In negative ion mode, 983 metabolites were detected in all samples, 49 differential metabolites were identified between the GDM and HPW groups (29 upregulated and 20 downregulated), 341 differential metabolites were identified between the GDM and HNPW groups (167 upregulated and 174 downregulated), and 245 differential metabolites were identified between the HPW and HNPW groups (112 upregulated and 133 downregulated). A total of nine differential metabolites with high confidence levels were identified in both the positive and negative ion modes, namely, L-isoleucine, D-glucose 6-phosphate, docosahexaenoic acid, arachidonic acid, adenosine, adenosine-monophosphate, adenosine 5′-monophosphate, xanthine, and hypoxanthine. Among all pathways enriched by the upregulated differential metabolites, the largest number of pathways were enriched by four differential metabolites, adenosine, adenosine 5′-monophosphate, D-glucose 6-phosphate, and adenosine-monophosphate, and among all pathways enriched by the downregulated differential metabolites, the largest number of pathways were enriched by three differential metabolites, L-isoleucine, xanthine, and arachidonic acid.

**Conclusion:**

Untargeted metabolomic analysis of saliva samples from pregnant women with GDM, HPW, and HNPW identified nine differential metabolites with high confidence. The results are similar to findings from previous metabolomics studies of serum and urine samples, which offer the possibility of using saliva for regular noninvasive testing in the population of pregnant women with and without GDM. Meanwhile, the associations between these identified differential metabolites and gingivitis need to be further validated by subsequent studies.

## 1 Introduction

It has been proven that periodontal disease is one of the risk factors for adverse pregnancy outcomes in pregnant women (Wu et al., 2015; Komine-Aizawa et al., 2019). Gingivitis is the most prevalent oral disease during pregnancy, which is more likely to occur in the second and third trimester of pregnancy. The gums of patients are hyperemic, swollen, and bleeding. Gingivitis affects 30%–70% of pregnant women worldwide (Dommisch et al., 2015), and the prevalence of pregnancy gingivitis is 60%–90% in China (Hu et al., 1999). Xiong et al. (2006) concluded that the prevalence of periodontitis in women with and without gestational diabetes mellitus (GDM) was 44.8% and 13.2%, respectively, and the results demonstrate a correlation between periodontitis and GDM. Therefore, it is clinically important to effectively prevent or treat periodontal diseases, control periodontitis-associated local and systemic inflammation, maintain oral health, and avoid the occurrence of adverse pregnancy outcomes in pregnant women, especially in pregnant women with GDM.

Saliva is a complex fluid that plays an important role in the maintenance of oral health. Salivary components contain not only a large amount of water, but also various electrolytes, proteins, and a large amount of volatile organic compounds that originate from compounds produced by microorganisms in the oral cavity, such as fatty ammonia, branched-chain fatty acids, indoles, phenols, and volatile sulfur-containing compounds (Cheng et al., 2016). Many blood components enter the saliva via intracellular transport pathways (passive intracellular diffusion and active transport) or paracellular pathways (extracellular ultrafiltration) (Haeckel and Hnecke, 1993; Jusko and Milsap, 1993). Most compounds found in blood are also present in saliva. Saliva testing can provide insight into the health and disease status of human body.

Untargeted metabolomics is an approach that aims to identify differentially expressed metabolites using univariate and multivariate statistical methods, thus reflecting the internal environment of cells and their interaction with external influencing factors. Goldsmith (Goldsmith et al., 2010) et al. suggest that metabolomics has an important role in clinical diagnosis of diseases. In recent years, a lot of attention has been paid to the basic saliva research; saliva presents an obvious advantage in diagnosing diseases earlier (Cheng et al., 2014; Zhang et al., 2016). Metabolomics full-spectrum analysis is a technique for the identification and quantification of all metabolites in organisms, discovering the relative relationship between metabolites and physiopathological changes, which focuses on small molecules with a relative molecular mass of less than 1,000, such as lipids, ketones, and organic acids.

Therefore, in this study, we conducted untargeted metabolomics on saliva samples from pregnant women with GDM, healthy pregnant women (HPW), and healthy non-pregnant women (HNPW) using liquid chromatography–tandem mass spectrometry (LC-MS/MS) to investigate the differences in salivary metabolites between these patients, and explore their possible associations with gingivitis, in an attempt to identify possible key metabolites and related metabolic pathways, and provide new ideas for the prevention and treatment of GDM and pregnancy gingivitis.

## 2 Materials and methods

### 2.1 Saliva samples

Twenty-seven pregnant and non-pregnant women who received preconception health examination and antenatal examination in the Maternal and Child Health Hospital of Changshou District in January 2022 were included, namely, nine pregnant women with GDM (mean age 28.9 ± 3.6 years, mean gestational age 30.1 ± 3.2 weeks, fasting plasma glucose 5.55 ± 0.17 mmol/L), nine HPW (mean age 27.9 ± 3.0 years, mean gestational age 28.6 ± 4.7 weeks, fasting plasma glucose 4.57 ± 0.33 mmol/L), and nine HNPW (mean age 27.7 ± 2.1 years, fasting plasma glucose 4.4 ± 0.085 mmol/L).

This study was approved by the Research Ethics Committee of Stomatological Hospital of Chongqing Medical University.

### 2.2 Inclusion criteria

Women were included in the GDM group if they were diagnosed with gestational diabetes mellitus (GDM) during antenatal examination by oral glucose tolerance test according to the diagnostic criteria recommended by the International Association of Diabetes and Pregnancy Study Group (IADPSG) in 2011; did not have dental caries; have moderate gingivitis assessed based on the modified Loe–Silness gingival index (presenting as shiny, red, swollen gums that bleed easily upon probing); had no systemic or congenital diseases, developmental malformations, and bacterial or severe infections in other parts of the body; did not take antibiotics, fluorides, and microecological modulators; and did not receive orthodontic treatment within the last 3 months.

Women were included in the HPW group if they did not have blood glucose abnormalities and dental caries; have moderate gingivitis assessed by the modified Loe–Silness gingival index (presenting as shiny, red, swollen gums that bleed easily upon probing); have no systemic or congenital diseases, developmental malformations, and bacterial or severe infections in other parts of the body; did not take antibiotics, fluorides, and microecological modulators; and did not receive orthodontic treatment within the last 3 months.

Women were included in the HNPW group if they did not have dental caries, gingivitis assessed by the modified Loe–Silness gingival index, systemic or congenital diseases, developmental malformations, and bacterial or severe infections in other parts of the body; and did not take antibiotics, fluorides, and microecological modulators, and did not receive orthodontic treatment within the last 3 months.

Patients with a history of chronic disease, diabetes, thyroid function disease, and other metabolic diseases are excluded from this study

### 2.3 Saliva sample collection

Non-stimulated whole saliva was collected from all participants according to the modified Rhodus method (Rhodus et al., 2005) between 9:00 and 11:00 a.m. All participants fasted 1 h prior to collection. During collection, participants were asked to let saliva collect in their mouth for at least 1 min and spit into a centrifuge tube or sterile cup; it is not allowed to spit sputum. This process needs to be repeated several times in order to ensure that an adequate volume (2–5 ml) of saliva was collected. The collected saliva samples were placed in an ice box and transported immediately to the laboratory. After centrifugation at 5,000*g* at 4°C for 10 min, the supernatant was collected and filtered through a 0.22-μm sterile membrane; 1 ml was dispensed into labeled 2-ml Eppendorf tubes and stored at −80°C. Before undertaking untargeted metabolomic analysis, all samples were taken and thawed.

Extraction and preparation steps of saliva metabolites: ① Add 100 µl of each sample into the corresponding centrifuge tube, and freeze the remaining samples. ② Add 700 µl of the extractant containing internal standard 1 (methanol:acetonitrile:water = 4:2:1), shake for 10 min, and place it in a refrigerator at −20°C for 2 h. ③ Centrifuge at 25,000*g* at 4°C for 15 min. ④ The sample is removed from the centrifuge and 600 µl of supernatant is transferred to a new centrifuge tube. ⑤ Drain with a drainer. ⑥ Add 180 µl of methanol:pure water (1:1 v/v) and swirl for 10 min until it is completely dissolved in the complex solution. ⑦ Centrifuge at 25,000*g* at 4°C for 15 min again. ⑧ The remaining samples (50 µl each) were taken into the three upper plates for the detection of positive and negative ions, and the other plate was used as the spare plate, and the remaining samples (20 µl mixed QC) were taken.

### 2.4 Untargeted metabolomic analysis of saliva samples

Untargeted metabolomic analysis of saliva samples from women with GDM, HPW, and HNPW was performed using the LC-MS/MS method. A high-resolution mass spectrometer, Q Exactive (Thermo Fisher Scientific, USA), was used to collect data in both the positive and negative ion modes in order to improve the coverage of metabolites. The raw data generated by LC-MS/MS were processed using Compound Discoverer 3.1 software (Thermo Fisher Scientific, USA) to perform peak alignment, peak picking, and compound identification.

### 2.5 Statistical analysis

Data pre-processing, statistical analysis, and metabolite taxonomic and functional annotations were performed using the metabolomics R software package metaX (Wen et al., 2017) and the metabolome information analysis process. Principal component analysis (PCA) was used to reduce the dimensionality of original multivariate data to analyze the groupings, trends (similarities and differences within and between sample groups), and outliers (presence of outlier samples) of the observed variables in the data set. The variable importance in the projection (VIP) values of the first two principal components of the partial least squares discriminant analysis (PLS-DA) model (Barker and Rayens, 2003; Westerhuis et al., 2008) combined with the multiplicity of variance change (fold change) obtained from the univariate analysis and the *t*-test (Student’s *t*-test) results were used to screen for differential metabolites.

In a strict sense, biological data did not strictly obey normal distribution. Before *t*-test, we processed the data by log2 to make the data approximately obey normal distribution, so that the result of *t*-test is relatively more reasonable. At the same time, considering that the difference between groups is not so significant, we used *p*-value (Zheng et al., 2019) as the condition for screening the difference in order to screen the appropriate differential metabolites for subsequent research and did not correct it.

## 3 Saliva metabolomics results

### 3.1 Results of sample quality control

**Figure 1:**
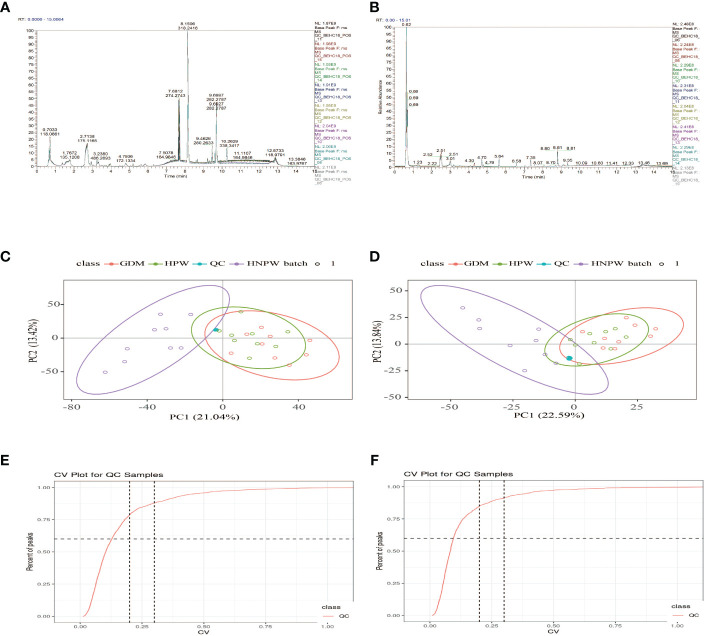
Base peak ion chromatograms of samples from each group. **(A)** Positive ion mode. **(B)** Negative ion mode; overlapping of base peak ion chromatograms of all the quality control samples showed that the chromatograms well overlapped in both the positive and negative ion modes, with small fluctuation in the retention time and peak response intensity, indicating that the instrument was in a good state with stable signal throughout the whole sample detection process. A PCA of the QC sample and all samples can be used to observe the overall distribution of each set of samples and the stability of the entire analytical process. As shown in **(C, D)**, the better the QC samples aggregate, the more stable the instrument and the better the repeatability of the acquired data. CV distribution of compounds in each sample as shown in **(E, F)**, and the number of compounds with a relative peak area CV of 30% or less in the QC sample. Ratio: The ratio of the number of compounds with a relative peak area CV less than or equal to 30% in the QC sample to the total number of compounds detected. Ratio ≥60%, the data quality is qualified. The two lines perpendicular to the *X*-axis in the figure are 20% and 30% CV reference line, and the line parallel to the *X*-axis is 60% of the reference line.

As shown in **Figures 1A, B**, after overlapping the base peak ion chromatograms of all quality control samples, the chromatograms well overlapped in both the positive and negative ion modes, the retention time and peak response intensity fluctuated little, indicating that the instrument was in a good state with stable signal during the whole sample detection process. A PCA analysis of the QC sample and all samples can be used to observe the overall distribution of each set of samples and the stability of the entire analytical process. As shown in **Figures 1C, D**, the better the QC samples aggregate, the more stable the instrument and the better the repeatability of the acquired data. CV distribution of compounds in each sample as shown in **Figures 1E, F**.

### 3.2 Results of compound identification

The results of this study showed that in positive ion mode, a total of 2,529 metabolites were detected in all saliva samples; 905 out of these 2,529 metabolites could be found in the Chemspider and mzCloud databases with corresponding compound information. In negative ion mode, a total of 983 metabolites were detected in all saliva samples, 335 out of these metabolites could be found in the Chemspider and mzCloud databases with corresponding compound information **Table 1**.

**Table 1:** Number of compounds and number of compounds with identification information identified in positive and negative ion modes.

Mode	Number of compounds	Number of compounds with identification information
Positive ion mode (pos)	2,529	905
Negative ion mode (neg)	983	335

Positive ion mode (pos): when the substances are ionized in an ion source, the adduct ions are positive ions, such as H^+^, ${\text{NH}}_{4}^{+}$ , Na^+^, and K^+^.

Negative ion mode (neg): when the substances are ionized in an ion source, the adduct ions are negative ions, such as −H, +Cl.

### 3.3 Classification of metabolites

**Figure 2:**
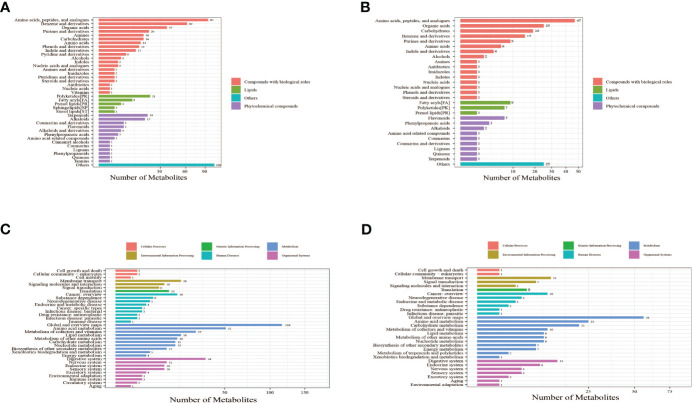
Bar chart of metabolite classification in positive ion mode **(A)** and negative ion mode **(B)**. The *X*-axis represents the number of metabolites in each class, and the *Y*-axis represents the metabolite classification entries. Others mean that classification information is the remaining category. The results showed that among the 905 molecular features identified in the positive ion mode, 515 molecular features were classified into four categories, including compounds with biological roles (*n* = 311), lipids (*n* = 38), phytochemical compounds (*n* = 61), and others (*n* = 105). Among the 335 metabolites identified in the negative ion mode, 126 molecular features were classified into four categories, including compounds with biological roles (*n* = 195), lipids (*n* = 17), phytochemical compounds (*n* = 18), and others (*n* = 25). Bar chart of KEGG functional annotation of metabolites in positive ion mode **(C)** and negative ion mode **(D)**. The *X*-axis represents the number of metabolites, and the *Y*-axis represents KEGG pathway entries. The results showed that in positive ion mode, 34 KEGG pathways involving 451 metabolites were annotated. The top four pathways with the largest number of metabolites were global and overview maps (116 metabolites), amino acid metabolism (51 metabolites), digestive system (34 metabolites), and metabolism of cofactors and vitamins (27 metabolites). The number of metabolites contained in these four pathways accounted for 50.55% of all metabolites annotated to the pathways. In negative ion mode, 30 KEGG pathways involving 238 metabolites were annotated. The top four pathways with the largest number of metabolites were global and overview maps (56 metabolites), amino acid metabolism (25 metabolites), carbohydrate metabolism (21 metabolites), and digestive system (13 metabolites). The number of metabolites contained in these four pathways accounted for 48.32% of all metabolites annotated to the pathways. These results suggest that metabolites with identification information detected and identified in either the positive or negative ion modes functioned mainly through two types of KEGG pathways, metabolism and organismal systems.

The identified metabolites were annotated using the Kyoto Encyclopedia of Genes and Genomes (KEGG) database and Human Metabolome Database (HMDB) to understand the classification of metabolites. The number of metabolites in each class is shown in **Figures 2A, B**. Identification results without classification information were not included in the analysis. At the same time, the identified metabolites were functionally annotated by the KEGG database in order to understand their functional properties, and determine the major biochemical metabolic pathways and signal transduction pathways involved in the metabolites. The number of metabolites identified in each type of metabolic pathways is shown **Figures 2C, D**.

### 3.4 Statistical analysis

**Figure 3:**
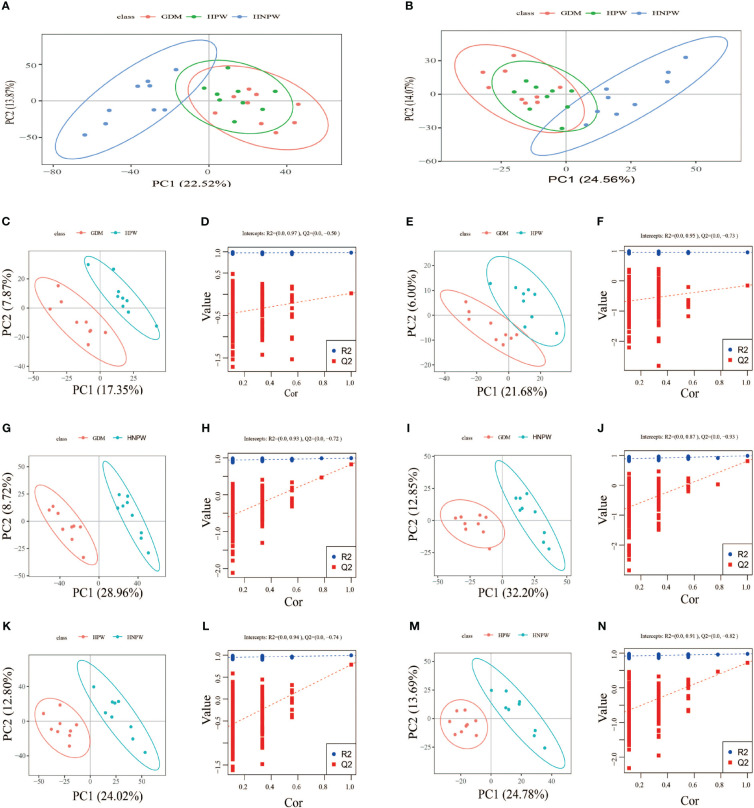
**(A, C, D, G, H, K, L)** Positive ion mode. **(B, E, F, I, J, M, N)** Negative ion mode. GDM, gestational diabetes mellitus; HPW, healthy pregnant women; HNPW, healthy non-pregnant women. A PCA model was constructed to observe the distribution and separation of samples between the groups. Data were log transformed (log2) prior to constructing PCA mode and scaled using the Pareto scaling method. PCA results. **(A, B)** The abscissa is the first principal component PC1, the ordinate is the second principal component PC2, and the ellipse in the PCA score graph is 95% confidence interval. Each dot represents a sample, and different groups are labeled with different colors. The number is the score of the principal component, which represents the percentage of the explanation on overall variance of the specific principal component. PLS-DA results. PLS-DA score plots between gestational diabetes mellitus (GDM) and healthy pregnant women (HPW) groups in positive **(C)** and negative ion modes **(E)**. PLS-DA score plots between the GDM group and the healthy non-pregnant women (HNPW) group in positive **(G)** and negative ion modes **(I)**. PLS-DA score plots between the HPW group and the HNPW group in positive **(K)** and negative ion modes **(M)**. The horizontal axis represents the first principal component; the vertical axis represents the second principal component. The number in parentheses is the score of the principal component, which represents the percentage of the overall variance explained by the corresponding principal component. Based on the following conditions, (1) the VIP of the first two principal components of the PLS-DA model ≥ 1; (2) fold-change ≥ 1.2 or ≤ 0.83; and (3) *p*-value< 0.05, the differential metabolites among groups were identified (**Table 2**). The two rightmost points in the figure are the actual R2Yand Q2 values of the PLS-DA model, and the remaining points are the R2Y and Q2 values obtained by randomly arranging the samples used **(D, F, H, J, L, N)**.

By comparing among groups in the positive ion mode, a total of 166 differential metabolites were identified between the GDM and HPW groups, of which 89 were upregulated and 77 were downregulated; a total of 823 differential metabolites were identified between the GDM and HNPW groups, of which 402 were upregulated and 421 were downregulated; a total of 647 differential metabolites were identified between the HPW and HNPW groups, of which 351 were upregulated and 296 were downregulated in the positive ion mode. In the negative ion mode, a total of 49 differential metabolites were identified between the GDM and HPW groups, of which 29 were upregulated and 20 were downregulated; a total of 341 differential metabolites were identified between the GDM and HNPW groups, of which 167 were upregulated and 174 were downregulated; a total of 245 differential metabolites were identified between the HPW and HNPW groups, of which 112 were upregulated and 133 were downregulated **Figure 3**.

**Table 2:** Differential metabolites among groups in positive and negative ion modes.

Group	Total number of differential metabolites	Number of up- and downregulated metabolites	Expression status	Number of metabolites with different confidence levels				
				Level 1	Level 2	Level 3	Level 4	Level 5
GDM vs. HPW in negative ion mode	49	29	Up	0	2	1	6	20
		20	Down	1	1	1	4	13
GDM vs. HPW in positive ion mode	166	89	Up	1	1	3	27	57
		77	Down	2	1	0	34	40
GDM vs. HNPW in negative ion mode	341	167	Up	4	6	0	43	115
		174	Down	0	6	1	36	131
GDM vs. HNPW in positive ion mode	823	402	Up	5	9	10	119	259
		421	Down	0	5	6	143	267
HPW vs. HNPW in negative ion mode	245	112	Up	8	5	1	68	30
		133	Down	0	6	0	26	101
HPW vs. HNPW in positive ion mode	647	351	Up	7	10	7	96	231
		296	Down	0	3	4	109	180

### 3.5 Results from volcano plot and metabolic pathway enrichment analysis of differential metabolites

**Figure 4:**
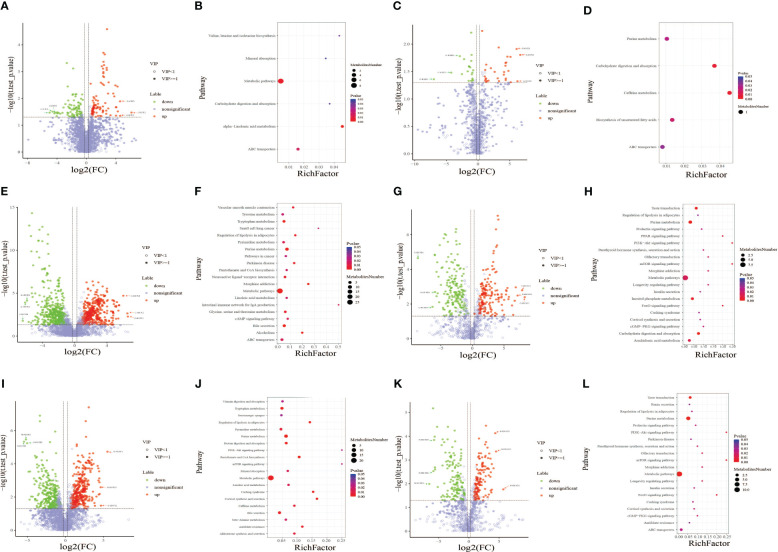
Volcano plot of differential metabolites between the gestational diabetes mellitus (GDM) and healthy pregnant women (HPW) groups in positive **(A)** and negative ion modes **(C)**. Volcano plot of differential metabolites between the GDM and healthy non-pregnant women (HNPW) groups in positive **(E)** and negative ion modes **(G)**. Volcano plot of differential metabolites between the HPW and HNPW groups in positive **(I)** and negative ion modes **(K)**. Green plots represent downregulated metabolites, red plots represent upregulated metabolites, and purple–gray plots represent meaningless metabolites. Bubble chart of KEGG enrichment analysis of differential metabolites identified between the gestational diabetes mellitus (GDM) and healthy pregnant women (HPW) groups in positive **(B)** and negative ion modes **(D)**. Bubble chart of KEGG enrichment analysis of differential metabolites identified between the GDM and healthy non-pregnant women (HNPW) groups in positive **(F)** and negative ion modes **(H)**. Bubble chart of KEGG enrichment analysis of differential metabolites identified between the HPW and HNPW groups in positive **(J)** and negative ion modes **(L)**.

Metabolic pathway enrichment analysis was performed based on the KEGG database **Figure 4**. Metabolic pathways with a *p*-value< 0.05 were considered to be significantly enriched by differential metabolites. The *X*-axis shows the enrichment factor. A larger enrichment factor indicates a greater degree of enrichment. The size of dots represents the number of differential metabolites annotated to the pathway. The dot size represents the number of differential metabolites annotated to this pathway. Enrichment analysis was based on annotated metabolites in the KEGG database. The annotation results of differentiated metabolites screened in this project were statistically analyzed by combining the hypergeometric test, and the *p*-value of corresponding pathway was obtained. Then, *p*-value< 0.05 was taken as the threshold to determine whether the pathway was enriched or not. The ggplot2 package in the R package is used for mapping.

In the present study, metabolic pathway enrichment analysis results of differential metabolites between GDM and HPW groups showed that in positive ion mode (**Figure 4B**), six enriched metabolic pathways were significantly different between the two groups, namely, alpha-linolenic acid metabolism [enriched by two metabolites 12-oxo phytodienoic acid and 13(s)-HOTrE]; valine, leucine, and isoleucine biosynthesis (enriched by L-isoleucine); carbohydrate digestion and absorption (enriched by sucrose); mineral absorption (enriched by L-isoleucine); ABC transporters (enriched by sucrose and L-isoleucine); and metabolic pathway [enriched by sucrose, hypoxanthine, L-isoleucine, dihydroxyindole, (+/-)-tropinone, bisphenol A, 12-oxo phytodienoic acid, and protoporphyrin IX]. In negative ion mode (**Figure 4D**), five enriched metabolic pathways were significantly different between the two groups, including caffeine metabolism (enriched by xanthine), carbohydrate digestion and absorption (enriched by maltotriose), and biosynthesis of unsaturated fatty acids (enriched by docosahexaenoic acid), purine metabolism (enriched by xanthine), and ABC transporters (enriched by maltotriose). Among the six differential metabolic pathways in positive ion mode, four differential pathways were enriched with L-isoleucine and three were enriched with sucrose. Among the five differential metabolic pathways in negative ion mode, two differential pathways were enriched with xanthine and two were enriched with maltotriose.

The results from metabolic pathway enrichment analysis of differential metabolites between GDM and HNPW groups showed that in positive ion mode (**Figure 4F**), 27 enriched metabolic pathways were significantly different between the two groups; the top three metabolic pathways with the largest differences between the two groups were intestinal immune network for IgA production (enriched with retinoate), small cell lung cancer (enriched with retinoate), and morphine addiction (enriched with two metabolites adenosine and dopamine). In negative ion mode (**Figure 4H**), 28 enriched metabolic pathways were significantly different between the two groups, and the top three differential metabolic pathways were mTOR and PI3K-Akt signaling pathways, FoxO and PPAR signaling pathways, and olfactory transduction, morphine addiction, and longevity regulating pathway; except for the PPAR signaling pathway that was enriched by 8(s)-hydroxy-(5z,9e,11z,14z)-eicosatetraenoic acid, the remaining pathways were enriched by adenosine 5′-monophosphate. Among the 29 differential metabolic pathways in the positive ionization mode, the largest number of metabolic pathways were enriched by the four differential metabolites adenosine, dopamine, arachidonic acid, and retinoate; these four metabolites were enriched in nine, eight, eight, and seven metabolic pathways, respectively. Among the 28 differential metabolic pathways in negative ion mode, the largest number of metabolic pathways were enriched by adenosine 5′-monophosphate and D-glucose 6-phosphate; these two metabolites were enriched in 19 and 7 metabolic pathways, respectively.

Metabolic pathway enrichment analysis results of differential metabolites between HPW and HNPW groups showed that in positive ion mode (**Figure 4J**), 27 enriched metabolic pathways were significantly different between the two groups; the top three metabolic pathways with the largest differences between the two groups were PI3K-Akt and mTOR signaling pathways, cortisol synthesis and secretion, and Cushing syndrome. Among these pathways, PI3K-Akt and mTOR signaling pathways were enriched by the differential metabolite adenosine-monophosphate, and the remaining pathways were enriched by adenosine-monophosphate and cortisol. In negative ion mode (**Figure 4L**), 27 enriched metabolic pathways were significantly different between the two groups; the top three differential metabolic pathways were PI3K-Akt and mTOR signaling pathways, FoxO signaling pathway, and olfactory transduction, morphine addiction, and longevity regulating pathway; these differential pathways were enriched by adenosine 5′-monophosphate. Among the 27 differential metabolic pathways in positive ion mode, the largest number of metabolic pathways were enriched by the five differential metabolites adenosine-monophosphate, arachidonic acid, L-threonine, L-methionine, and cortisol; these five metabolites were enriched in 10, 7, 6, 5, and 5 pathways, respectively. Among the 27 differential metabolic pathways in negative ion mode, the largest number of metabolic pathways were enriched by two differential metabolites, adenosine 5′-monophosphate and D-glucose 6-phosphate, which were involved in 20 and 5 pathways, respectively.

The results (**Tables 3**–**5**) showed that among the top three upregulated differential metabolites, only one metabolite with molecular formula C_18_H_1_ClN_2_O_6_S_2_ that can be found in ChemSpider and mzCloud databases (ID 187436) was classified into others, and belonged to the class of benzodioxoles, but this metabolite was not annotated to a pathway. Among the top three downregulated differential metabolites, one metabolite with molecular formula C_17_H_22_O_5_ that can be found in ChemSpider and mzCloud databases (ID 37260) was classified into phytochemical compounds, and belonged to the class of terpenoids; one metabolite (ChemSpider ID and mzCloud ID: 37260, molecular formula C_16_H_19_NO) was classified as benzene and derivatives, and had compounds with biological roles; and one metabolite (ChemSpider ID and mzCloud ID: 12665, molecular formula C_9_H_17_NO) was classified into others, and belonged to the class of piperidinones; these 3 metabolites were also not annotated to specific metabolic pathways. The results (**Tables 6**–**8**) showed that among the top three upregulated metabolites, only one metabolite (ChemSpider ID and mzCloud ID:30778505, molecular formula C_9_H_7_NO_5_S) was classified as indole and derivatives, had compounds with biological roles, and was not annotated to specific pathways. Among the top three downregulated metabolites, one metabolite with molecular formula C_15_H_29_NO_3_ (ChemSpider ID mzCloud code 21513291) and one metabolite with molecular formula C_12_H_11_NO_5_ (ChemSpider ID mzCloud code 74852585) were classified as amino acids, peptides, and analogues, and had compounds with biological roles, which were not annotated to specific metabolic pathways **Table 9**.

**Table 3:** Top 3 (pu1–pu3) upregulated and top 3 (pd1–pd3) downregulated differential metabolites between the gestational diabetes mellitus and healthy pregnant women groups in positive ion mode.

	Molecular formula	Molecular weight	Level	Family	Metabolites
D-P pu1	C_12_ H_25_NO_11_	359.142 Da	Level 5	–	–
D-P pu2	C_18_H_1_ClN_2_O_6_S_2_	454.0046 Da	Level 4	Benzodioxoles	Other
D-P pu3	–	159.0301 Da	Level 5	–	–
D-P pd1	C_13_H_22_N_6_O_3_	310.1756 Da	Level 5	–	–
D-P pd2	C_19_H_29_N_5_O_2_	359.2308 Da	Level 4	–	–
D-P pd3	C_17_H_22_O_5_	306.1467 Da	Level 4	Terpenoids	Phytochemical compounds

**Table 4:** Top 3 (pu1–pu3) upregulated and top 3 (pd1–pd3) downregulated differential metabolites between the gestational diabetes mellitus and healthy non-pregnant women groups in positive ion mode.

	ChemSpider ID mzCloud	Molecular formula	Molecular weight	Level	Family	Metabolites
D-W pu1	–	–	220.0349 Da	Level 5	–	–
D-W pu2	–	–	379.0877 Da	Level 5	–	–
D-W pu3	–	–	261.0616 Da	Level 5	–	–
D-W pd1	12665	C_9_H_17_NO	155.1312 Da	Level 4	Piperidinones	Others
D-W pd2	37260	C_16_H_19_NO	241.1467 Da	Level 4	Benzene and derivatives	Compounds with biological roles
D-W pd3	59352	C_33_H_34_N_4_O_6_	582.2484 Da	Level 4	–	–

**Table 5:** Top 3 (pu1–pu3) upregulated and top 3 (pd1–pd3) downregulated differential metabolites between the healthy pregnant women and healthy non-pregnant women groups in positive ion mode.

	ChemSpider ID mzCloud	Molecular formula	Molecular weight	Level	Family	Metabolites
P-W pu1	–	C_7_H_12_N_5_O_4_P	261.0616 Da	Level 5	–	–
P-W pu2	29738718	C_24_H_19_FN_2_O_3_	402.1362 Da	Level 4	–	–
P-W pu3	–	C_10_H_22_NO_12_P	379.0877 Da	Level 5	–	–
P-W pd1	–	C_26_H_46_N_6_O_9_	586.3327 Da	Level 5	–	–
P-W pd2	6001	C_17_H_12_I_2_O_3_	517.8899 Da	Level 4	–	–
P-W pd3	7826270	C_27_H_32_F6O_3_	518.2244 Da	Level 4	–	–

**Table 6:** Top 3 (pu1–pu3) upregulated and top 3 (pd1–pd3) downregulated differential metabolites between the gestational diabetes mellitus and healthy pregnant women groups in negative ion mode.

	ChemSpider ID mzCloud	Molecular formula	Molecular weight	Level	Family	Metabolites
D-P nu1	–	C_14_H_19_N_5_O_7_S	401.0987 Da	Level 5	–	–
D-P nu2	–	C_16_H_24_N_4_O_10_	432.1473 Da	Level 5	–	–
D-P nu3	–	C_34_H_62_N_6_O_8_	682.4654 Da	Level 5	–	–
D-P nd1	21513291	C_15_H_29_NO_3_	271.2142 Da	Level 4	Amino acids, peptides, and analogues	Compounds with biological roles
D-P nd2	–	C_6_H_5_C_l_O_3_S	191.9647 Da	Level 5	–	–
D-P nd3	74852585	C_12_H_11_NO_5_	249.0633 Da	Level 4	Amino acids, peptides, and analogues	Compounds with biological roles

**Table 7:** Top 3 (pu1–pu3) upregulated and top 3 (pd1–pd3) downregulated differential metabolites between the gestational diabetes mellitus and healthy non-pregnant women groups in negative ion mode.

	ChemSpider ID mzCloud	Molecular formula	Molecular weight	Level	Family	Metabolites
D-W nu1	30778505	C_9_H_7_NO_5_S	241.0042 Da	Level 4	Indole and derivatives	Compounds with biological roles
D-W nu2	–	C_7_H_4_N_2_O_4_S	211.989 Da	Level 5	–	–
D-W nu3	–	C_20_H_37_N_3_O_10_	479.2471 Da	Level 5	–	–
D-W nd1	74852585	C_12_H_11_NO_5_	249.0633 Da	Level 4	Amino acids, peptides, and analogues	Compounds with biological roles
D-W nd2	–	C_46_H_75_N_4_O_16_P	970.4906 Da	Level 5	–	–
D-W nd3	–	C_23_H_41_N_5_O_7_	499.2996 Da	Level 5	–	–

**Table 8:** Top 3 (pu1–pu3) upregulated and top 3 (pd1–pd3) downregulated differential metabolites between the healthy pregnant women and healthy non-pregnant women groups in negative ion mode.

	ChemSpider ID mzCloud	Molecular formula	Molecular weight	Level	Family	Metabolites
P-W nu1	–	C_7_H_4_N_2_O_4_S	211.989 Da	Level 5	–	–
P-W nu2	–	C_17_H_32_N_2_O_8_	392.2155 Da	Level 5	–	–
P-W nu3	–	C_35_H_60_N_4_O_13_	744.4185 Da	Level 5	–	–
P-W nd1	–	C_46_H_75_N_4_O_16_P	970.4906 Da	Level 5	–	–
P-W nd2	–	C_26_H_46_N_6_O_9_	586.3324 Da	Level 5	–	–
P-W nd3	65099	C_27_H_50_O_7_P_2_	548.3044 Da	Level 4	–	–

**Table 9:** Enrichment of metabolic pathways of differential metabolites with identification confidence levels 1 and 2 between different groups.

Group	Model	Location	Name	Level	KEGG ID	Pathway ID	Family	Metabolites
GDM vs. HPW	Pos	Down 54/77	L-isoleucine	Level 1	C00407	map01100 Metabolic pathways map02010 ABC transporters map00290 Valine, leucine, and isoleucine biosynthesis map04978 Mineral absorption	Amino acids	Compounds with biological roles
		Down 66/77	Hypoxanthine	Level 1	C00262	map01100 Metabolic pathways	Purines and derivatives	Compounds with biological roles
	Neg	Down 8/20	Docosahexaenoic acid	Level 2	C06429	map01040 Biosynthesis of unsaturated fatty acids	Fatty acyls	Lipids
		Down 14/20	Xanthine	Level 1	C00385	map00230 Purine metabolism map00232 Caffeine metabolism	Purines and derivatives	Compounds with biological roles
GDM vs. HNPW	Pos	Up 7/402	Adenosine	Level 2	C00212	map00230 Purine metabolism map01100 Metabolic pathways map04024 cAMP signaling pathway map04080 Neuroactive ligand–receptor interaction map04270 Vascular smooth muscle contraction map04923 Regulation of lipolysis in adipocytes map05012 Parkinson disease map05032 Morphine addiction map05034 Alcoholism	Purines and derivatives	Compounds with biological roles
		Down 213/421	Retinoate	Level 2	C00777	map01100 Metabolic pathways map04659 Th17 cell differentiation map04672 Intestinal immune network for IgA production map05200 Pathways in cancer map05222 Small cell lung cancer map05223 Non-small cell lung cancer map05226 Gastric cancer	Prenol lipids	Lipids
		Down 271/421	Arachidonic acid	Level 2	C00219	map00591 Linoleic acid metabolism map01100 Metabolic pathways map04270 Vascular smooth muscle contraction map04726 Serotonergic synapse map04750 Inflammatory mediator regulation of TRP channels map04912 GnRH signaling pathway map04923 Regulation of lipolysis in adipocytes map05140 Leishmaniasis	Fatty acyls	Lipids
	Neg	Up 7/167	Adenosine 5'-monophosphate	Level 2	C00020	map00230 Purine metabolism map01100 Metabolic pathways map01523 Antifolate resistance map04022 cGMP-PKG signaling pathway map04068 FoxO signaling pathway map04150 mTOR signaling pathway map04151 PI3K-Akt signaling pathway map04152 AMPK signaling pathway map04211 Longevity regulating pathway map04740 Olfactory transduction map04742 Taste transduction map04923 Regulation of lipolysis in adipocytes map04924 Renin secretion map04925 Aldosterone synthesis and secretion map04927 Cortisol synthesis and secretion map04928 Parathyroid hormone synthesis, secretion and action map04934 Cushing syndrome map05012 Parkinson disease map05032 Morphine addiction	Purines and derivatives	Compounds with biological roles
		Up 117/167	D-glucose 6-phosphate	Level 2	C00092	map00562 Inositol phosphate metabolism map01100 Metabolic pathways map04911 Insulin secretion map04917 Prolactin signaling pathway map04918 Thyroid hormone synthesis map04931 Insulin resistance map04973 Carbohydrate digestion and absorption	Carbohydrates	Compounds with biological roles
		Down 174/174	8(s)-hydroxy-(5z,9e,11z,14z)-eicosatetraenoic acid	Level 2	C14776	map00590 Arachidonic acid metabolism map01100 Metabolic pathways map03320 PPAR signaling pathway	Fatty acyls	Lipids
HPW vs. HNPW	Pos	Up 189/351	Adenosine-monophosphate	Level 1	C00020	map00230 Purine metabolism map01100 Metabolic pathways map01523 Antifolate resistance map04068 FoxO signaling pathway map04150 mTOR signaling pathway map04151 PI3K-Akt signaling pathway map04923 Regulation of lipolysis in adipocytes map04925 Aldosterone synthesis and secretion map04927 Cortisol synthesis and secretion map04934 Cushing syndrome	Nucleic acids	Compounds with biological roles
		Up 192/351	L-(−)-methionine	Level 2	C00073	map00970 Aminoacyl-tRNA biosynthesis map01100 Metabolic pathways map01523 Antifolate resistance map04974 Protein digestion and absorption map04978 Mineral absorption	Amino acids	Compounds with biological roles
		Up 235/351	Cortisol	Level 2	C00735	map01100 Metabolic pathways map04927 Cortisol synthesis and secretion map04934 Cushing syndrome map04976 Bile secretion	Steroids and derivatives	Compounds with biological roles
		Down 93/296	Arachidonic acid	Level 2	C00219	map00591 Linoleic acid metabolism map01100 Metabolic pathways map04726 Serotonergic synapse map04912 GnRH signaling pathway map04923 Regulation of lipolysis in adipocytes map04925 Aldosterone synthesis and secretion map05140 Leishmaniasis	Fatty acyls	Lipids
	Neg	Up 9/112	Adenosine 5'-monophosphate	Level 2	C00020	map00230 Purine metabolism map01100 Metabolic pathways map01523 Antifolate resistance map04022 cGMP-PKG signaling pathway map04024 cAMP signaling pathway map04068 FoxO signaling pathway map04150 mTOR signaling pathway map04151 PI3K-Akt signaling pathway map04152 AMPK signaling pathway map04211 Longevity regulating pathway map04740 Olfactory transduction map04742 Taste transduction map04923 Regulation of lipolysis in adipocytes map04924 Renin secretion map04925 Aldosterone synthesis and secretion map04927 Cortisol synthesis and secretion map04928 Parathyroid hormone synthesis, secretion and action map04934 Cushing syndrome map05012 Parkinson disease map05032 Morphine addiction	Purines and derivatives	Compounds with biological roles
		Up 93/112	D-glucose 6-phosphate	Level 2	C00092	map01100 Metabolic pathways map04911 Insulin secretion map04917 Prolactin signaling pathway map04918 Thyroid hormone synthesis map04931 Insulin resistance	Carbohydrates	Compounds with biological roles

### 3.6 Metabolic pathway enrichment analysis results of differential metabolites with identification confidence levels 1 and 2 between different groups

**Table 9** presents the specific information of each differential metabolite with confidence levels 1 and 2, after conducting pathway enrichment analysis of (GDM vs. HPW), (GDM vs. HNPW), and (HPW vs. HNPW) differential metabolites.

## 4 Discussion

In the present study, nine samples were included in each group. Based on our previous study on oral microbial diversity of dental plaque and salivary samples from nine pregnant women with GDM, nine HPW, and nine HNPW, at both species and genus levels, species accumulation curves showed that when the sample size reached nine per group, the number of new species in different oral environments would not significantly increase with an increasing sample size, there is a tendency toward saturation in species richness, and all currently recognized pathogenic bacteria associated with periodontal diseases have been detected. Therefore, in the present study, we considered a sample size of nine for each group, and performed untargeted metabolomic analysis to identify salivary metabolites that were differentially expressed in the saliva of women with GDM, HPW, and HNPW, and explore the possible correlation between the identified differential salivary metabolites and periodontal health.

The combination of three dimensions, retention time, and MS1 and MS2 spectra, is currently the most widely used approach to improve the confidence of metabolite identification in metabolomic analysis (Liang et al., 2020; Shen et al., 2020). In terms of confidence levels of metabolites identified in the present study, among the top three upregulated and top three downregulated differential metabolites identified in both the positive and negative ion modes (36 metabolites), 14 metabolites were identified with identification confidence level 4 and 22 were identified with identification confidence level 5. It can be seen that although a variety of differential metabolites were identified, relatively few metabolites with high confidence were available for further analysis, and their biological information needs to be further explored and analyzed.

Furthermore, we can see that although the metabolic pathway is enriched by the largest number of differential metabolites, the pathway showed little difference between groups.

Branched-chain amino acids (BCAAs) are essential amino acids, including leucine, isoleucine, and valine, which cannot be synthesized by the human body itself. The levels of BCAAs in plasma are associated with diabetes. The results of this study showed that compared with the HNPW group, the level of isoleucine was decreased in the GDM group, and increased in the HPW group, but the differences were not statistically significant, whereas there were statistically significant differences between the GDM and HPW groups. Since isoleucine cannot be synthesized endogenously, and needs to be absorbed exogenously, the above-mentioned results indicate that the absorption of isoleucine was obviously reduced in pregnant women with GDM compared to HPW. In terms of differential metabolic pathways involving L-isoleucine between the GDM and HPW groups, we found that except for the shared differential metabolic pathways, ABC transporters; Valine, leucine, and isoleucine biosynthesis; and mineral absorption were differential metabolic pathways between the two groups. A previous study has shown that the ABC transporter family is associated with the development and progression of diabetes, dietary isoleucine can be absorbed through the intestine to bypass the hepatic first pass effect (Mann et al., 2021), while the P-glycoprotein (P-gp) encoded by the ABCB1 gene is mainly distributed in specific tissues such as the intestine, kidney, liver, and cerebrovascular endothelium, and the function and expression of P-GP are altered under diabetic conditions (Liu et al., 2006; Liu et al., 2007; Liu et al., 2008). Meanwhile, ABCC8 and ABCC9 are important components of ATP-sensitive potassium (KATP) channels, which can regulate KATP channel activity, and modulate insulin release to control blood glucose levels (Aguilar-Bryan and Bryan, 1999; Bryan et al., 2007). Therefore, we speculate that the changes in isoleucine level in patients with GDM may be caused by changes in P-gp, ABCC8, and ABCC9 expression.

Numerous studies (Doi et al., 2005; Doi et al., 2007; Ikehara et al., 2008; Guasch-Ferré et al., 2016; Ullrich et al., 2016; Newmire et al., 2019; Elovaris et al., 2021a; Elovaris et al., 2021b) have suggested that the effect of isoleucine on glucose metabolism may be related to the decreased expression of glucose-6-phosphatase (G6Pase). In the present study, we found that glucose 6-phosphate (G6P) level was elevated after pregnancy, presumably due to a decrease in G6Pase expression. Additionally, we found that except for the shared differential metabolic pathways involving D-glucose 6-phosphate, two pathways, namely, inositol phosphate metabolism and carbohydrate digestion and absorption, differed significantly between the GDM and HNPW groups. However, the changes in these two differential metabolic pathways did not result in a significant difference in changes in G6P level between the GDM and HPW groups. From these results, we hypothesize that changes in isoleucine expression, either upregulation or downregulation, can both lead to a decrease in G6Pase expression. These results are similar to the findings from the previous studies investigating the alterations of isoleucine in diabetes.

Insulin resistance is a predominant pathogenic component of GDM. Several population studies have found that GDM can cause changes in maternal fatty acid metabolism, especially polyunsaturated fatty acids (PUFAs), during the third trimester of pregnancy (Wijendran et al., 1999; Thomas et al., 2004; Chen et al., 2010). PUFAs can be classified into n-3 PUFA [mainly derived from eicosapentaenoic acid (EPA), docosahexaenoic acid (DHA), and α-linolenic acid (ALA)] and n-6 PUFA [mainly derived from linoleic acid (LA) and arachidonic acid (AA)]. ALA is a dietary precursor for EPA and DHA (Gogus and Smith, 2010) and exerts anti-inflammatory and immunomodulatory effects mainly through regulation of cell proliferation and response activity, production of inflammatory cytokines, and adhesion molecule expression (Ho et al., 2011; Wutzler et al., 2013; Liu et al., 2015). LA, a precursor in the synthesis of AA, can increase the risk of chronic diseases via regulating inflammatory responses (Simopoulos, 2003; Yary et al., 2016).

The synthesis of other long-chain polyunsaturated fatty acids (LC-PUFAs) of the same series requires the enzyme systems such as fatty acid desaturases and elongase enzymes. The n-3 and n-6 PUFA synthesis share the same set of enzymes, resulting in the occurrence of competitive inhibition (Mariamenatu and Abdu, 2021). The mechanism linking the ratio of n-6 and n-3 PUFAs and diabetes has not yet been clarified. One explanation is that n-3 and n-6 PUFAs compete for desaturase and elongase enzymes, and conversion of LA to AA and ALA to DHA and EPA occurs through desaturation and elongation by δ-6 and δ-5 desaturases (Mozaffarian et al., 2005). ALA and its metabolites can inhibit the conversion of LA to AA, thus reducing the production of inflammatory markers (Pischon et al., 2003). However, a higher intake of ALA may affect the pathway of n-6 PUFA metabolism. PUFAs can be transported from the mother to the fetus through the placenta (Makrides et al., 1994; Salem et al., 1996; Greiner et al., 1997), and the placenta preferentially takes up and transports fatty acids essential for fetal growth and development (Haggarty et al., 1997; Haggarty et al., 1999), with the order of preference being AA > DHA > ALA > LA (Haggarty, 2002).

GDM causes metabolic disorders involving fatty acids in maternal and cord blood, but fatty acid alterations display different trends in different tissues; for example, DHA levels are markedly higher in the serum or plasma and lower in the erythrocyte membranes of patients with GDM; these differences in AA and DHA levels in the serum/plasma and the erythrocyte membranes may be related to the negative feedback regulation of the human body. The plasma fatty acids reflect short-term fatty acid intake (1 to 2 weeks), and the plasma fatty acid levels are influenced by many factors, such as the physiological state of the body, dietary intake, and genes, whereas the erythrocyte fatty acid levels can accurately reflect the long-term (approximately 1 to 2 months) dietary fatty acid intake (Hai-Tao et al., 2021).

The results of this study showed that DHA expression was significantly downregulated in the GDM group compared with the HPW group. Meanwhile, DHA expression was significantly downregulated in the GDM group compared with the HNPW group, whereas no statistically significant difference was found between the HPW and HNPW groups. DHA was annotated to biosynthesis of unsaturated fatty acids (map01040) pathway. Compared with the HNPW group, AA levels was significantly downregulated in both the GDM and HPW groups, but no statistically significant difference was found between GDM and HPW groups. In terms of differential metabolic pathways involving AA, changes in AA expression in two pathways including vascular smooth muscle contraction map (map04270) and inflammatory mediator regulation of TRP channels (map04750) were observed in the GDM group when compared with the HNPW group. Unlike the GDM group, changes in AA expression were observed in aldosterone synthesis and secretion (map04925) pathway in the HPW group when compared with the HNPW group.

We speculate that the possible reasons for the downregulation of DHA in the GDM group are as follows: (i) the lack of precursor substances for DHA synthesis due to inadequate ALA intake from foods in GDM patients; (ii) although adequate ALA intake from foods is achieved, the synthesis of AA is stronger than that of DHA due to the presence of insulin resistance and the competition between n-3 and n-6 PUFAs. According to the differential metabolic pathways involving AA observed in this study, we suggest that when GDM occurs, AA is involved in the chronic inflammatory response, and has an impact on the placental transport of maternal PUFAs.

Under normal physiological conditions, the ratio of adenosine monophosphate (AMP), adenosine bisphosphonate (ADP), and adenosine triphosphate (ATP) is in a relatively stable state. However, under excessive starvation, ischemic conditions, or other extreme conditions, the production of ATP is insufficient, ADP accumulation occurs, and the lack of ATP is compensated to some extent through the reaction 2ADP → ATP + AMP, resulting in an increase in AMP levels. Since the AMP/ATP ratio varies as the square of the ADP/ATP ratio (Hardie and Hawley, 2001), sensing the levels of AMP is more sensitive than ADP.

When AMP content is high with low energy, the phosphorylation of AMP-activated protein kinase (AMPK) by the upstream kinases is promoted, thus increasing AMPK activity (Hawley et al., 1995; Xiao et al., 2007). Allosteric activation of AMPK induced by AMP further results in a two- to threefold increase in AMPK activity after phosphorylation, the increase varies with ATP levels (Gowans et al., 2013), and maximum AMPK activation can be reached (Sanders et al., 2007). AMP and ADP binding also inhibits AMPK dephosphorylation mediated by phosphatases (Davies et al., 1995; Xiao et al., 2011), and the binding of AMPK complexes to ADP or AMP also leads to conformational changes, thus promoting phosphorylation of a threonine residue (Thr-172) and inhibiting its dephosphorylation. AMPK activation can improve insulin sensitivity and glucose homeostasis, and AMPK inactivation is associated with various metabolic disorders, reflecting its importance as a therapeutic target (Cabarcas et al., 2010).

AMP is an important regulator of insulin and Akt protein kinase signaling pathways. It has been shown that Akt could regulate the inhibitory effect of insulin on AMPK (Kovacic et al., 2003). Akt cannot directly phosphorylate AMPK. Insulin-induced changes in Akt activity can regulate AMPK activity by altering the intracellular AMP/ATP ratio. In fact, the activation of Akt could reduce the intracellular AMP/ATP ratio, leading to a decrease in AMPK activity (Hahn-Windgassen et al., 2005). Therefore, the insulin–Akt signaling axis can expand the range of metabolic effects by upregulating AMP signaling and increasing AMPK activity.

The results of the present study showed that compared with the HNPW group, the expression of adenosine and adenosine 5′-monophosphate was significantly upregulated in the GDM group, and the expression of adenosine-monophosphate and adenosine 5′-monophosphate was significantly upregulated in the HPW group, but the difference was not significant between the GDM and HPW groups. In terms of the annotated differential metabolic pathways, except for the cAMP signaling pathway involving adenosine 5′-monophosphate that was determined in the HPW group when compared with the HNPW group, differential metabolic pathways of the GDM and HNPW groups compared with the HNPW group were the same. This also seems to indicate that that, after pregnancy, the energy requirements of pregnant women increase, and the AMPK activity changes accordingly, so it is speculated that insulin sensitivity and glucose homeostasis in GDM patients may be affected if the changes in AMPK activity are insufficient to meet the body’s needs.

GDM and type 2 diabetes mellitus have similar precipitating factors leading to glucose metabolism disorders. Uric acid has been suggested to possibly affect oxidative stress, inflammatory responses, and enzymes associated with glucose and lipid metabolic homeostasis (Lima et al., 2015). The results of this study showed that uric acid expression was significantly upregulated in both the GDM and HPW groups compared with the HNPW group, whereas no statistically significant difference was found between the GDM and HPW groups. However, the expression of uric acid precursors, anthine and hypoxanthine, was significantly downregulated in the GDM group compared with the PWM group; there was no statistically significant difference between the GDM and HNPW groups. In terms of differential metabolic pathways involving anthine and hypoxanthine among GDM and HPW groups, xanthine was annotated to two pathways, purine metabolism (map00230) and caffeine metabolism (map00232), and hypoxanthine was only annotated to metabolic pathways (map01100), but the next level of pathways is not yet known.

At present, according to findings from previously published studies on the relationship between serum uric acid levels and GDM, some scholars suggest that uric acid levels were positively correlated with the risk of GDM (Kharb, 2008; Laughon et al., 2009; Wolak et al., 2010; Wang, 2012; Gkiomisi et al., 2013; Rasika et al., 2014; Aker et al., 2016; Amudha et al., 2017), while some studies documented that there was no significant difference in the uric acid levels between patients with GDM and healthy controls (Seghieri et al., 2003; Güngör et al., 2006; Davari-Tanha et al., 2008; Maged et al., 2014), and some even suggest that serum uric acid levels were significantly lower in the GDM group than in the non-GDM group (Javadian et al., 2014). The results of a meta-analysis conducted by Zhao support a correlation between uric acid levels and the incidence of GDM (Diqi, 2018).

Xanthine oxidoreductase (XOR) has two redox isoforms, xanthine dehydrogenase (XDH) and xanthine oxidase (XO), which is mainly found in capillary endothelial cells, and these two forms are interconvertible (Battelli et al., 2014). Under normal physiological conditions, it mainly exists in the form of XDH. During ischemia and hypoxia, the synthesis of XDH increases due to the decrease of ATP production and the dysfunction of membrane pump, which is converted to large amounts of XO. At the same time, ATP cannot be used to release energy, and is degraded to ADP, AMP, and hypoxanthine, leading to large accumulation of hypoxanthine in the ischemic tissues. During reperfusion, a large amount of molecular oxygen enters into the ischemic tissues along with the blood, XO catalyzes the conversion of hypoxanthine to xanthine again, and further catalyzes the conversion of xanthine to uric acid. These processes use molecular oxygen as an electron acceptor, resulting in the production of a large amount of superoxide anion and hydrogen peroxide (Nørholt et al., 1996; Chen et al., 2004), which can cause hypoxic tissue damage directly, and the body may also enter a state of oxidative stress, leading to vascular endothelial damage and promoting the progression of GDM.

Based on the findings of the above-mentioned previous studies and results of the present study, we hypothesized that the elevated expression of xanthine and hypoxanthine in the HPW group may be due to the relatively lower degree of hypoxia in the HPW group compared to the GDM group; this leads to a decrease in the production or activity of XO, thereby decreasing the synthesis of uric acid. Additionally, the oxygen demand is higher in patients with GDM than in HPW.

**Figure 5:**
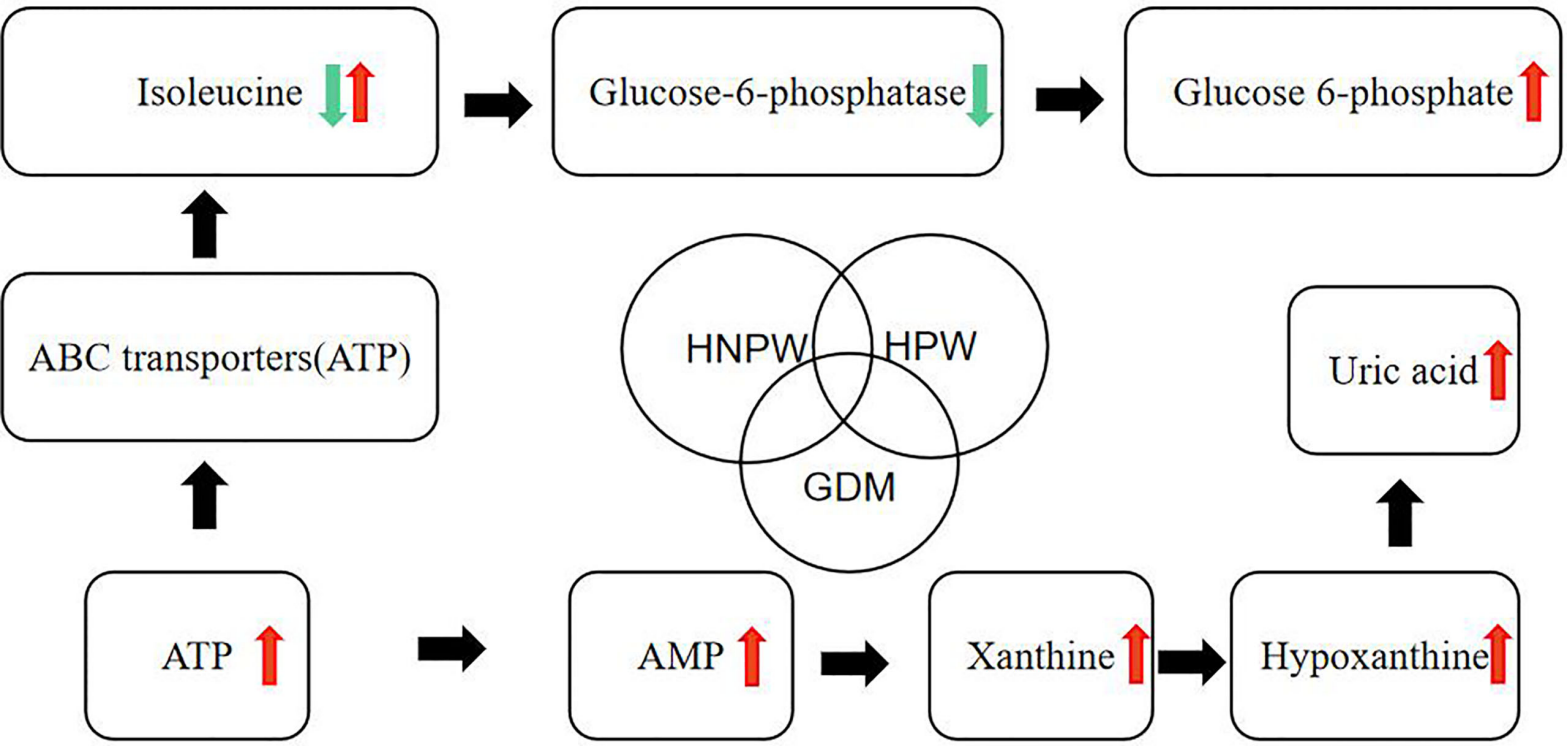
Hypothetical diagram about differential metabolites.

In this study, we made a hypothetical map (**Figure 5**) of several metabolites that were found to be different in the comparison of GDM, HPW, and HNPW, so as to further elaborate and confirm their correlation in subsequent studies.

## 5 Conclusion

In conclusion, untargeted metabolomic analysis of saliva samples from pregnant women with DGM, HPW, and HNPW identified nine differential metabolites with high confidence. The results are similar to findings from previous metabolomics studies of serum and urine samples, which offer the possibility of using saliva for regular noninvasive testing in the population of pregnant women with and without GDM. Meanwhile, the associations between these identified differential metabolites and gingivitis need to be further validated by subsequent studies.

## Data availability statement

The data presented in the study are deposited in the MetaboLights database repository, accession number MTBLS7774.

## Ethics statement

The studies involving human participants were reviewed and approved by The Research Ethics Committee of Stomatological Hospital of Chongqing Medical University. The patients/participants provided their written informed consent to participate in this study.

## Author contributions

YL, YF, and ZY conceived the research theme and supervised the entire study. YL collected the data, analyzed the data, drew the figures, explained the results, and drafted the manuscript. DJ, JL, and ZZ revised the manuscript and performed reference collection. All authors contributed to the article and approved the submitted version.
